# Abscopal effect leading to complete disappearance of extensive meningiomatosis after gamma knife radiosurgery: Case report

**DOI:** 10.3389/fsurg.2022.908645

**Published:** 2022-07-14

**Authors:** Salman Aldakhil, David Mathieu

**Affiliations:** Surgery/Neurosurgery, Université de Sherbrooke, Sherbrooke, QC, Canada

**Keywords:** abscopal effect, case report, meningioma, gamma knife, stereotactic radiosurgery

## Abstract

**Background and Importance:**

The abscopal effect is partial or complete tumor response in a separate site that was not the target of prior local treatment. There have been only 50 well-documented cases from 1960 to 2014. Our case is the first one of presumed low-grade meningioma demonstrating a possible response *via* the abscopal effect after single-fraction stereotactic radiosurgery.

**Clinical Presentation:**

A case of a 70-years-old female with extensive intracranial meningiomatosis who had complete disappearance of all tumors after gamma knife radiosurgery targeting the right petroclival part of the tumor. She had complete resolution of her symptoms, which included hearing loss, headache, ataxia and dysphagia.

**Conclusion:**

The abscopal effect is an extremely rare phenomenon after local radiation therapy of a remote tumor. Our case demonstrates that this can occur in remote meningiomas after single fraction GKRS of a different tumor.

## Introduction

The therapeutic effect of radiation on tumor is complex and involves many different mechanisms, which include apoptosis due to direct and indirect DNA damage, mitotic catastrophe leading to cellular senescence, lipid peroxidation and iron-dependent cell death, among others (1). Radiation can also induce an immune-mediated response in the irradiated tumor, that can be amplified with concurrent immunotherapy. A phenomenon called the abscopal effect has been described as a rare occurrence after radiation therapy. This is defined by partial or complete tumor response in a separate site that was not the target of prior local treatment. It was first described by Mole in 1953 (2) and there has been only 50 well-documented cases from 1960 to 2014 (3). The exact reason behind this effect is not known with certainty yet, but most authors believe remote activation of immunological pathways have an important role. In this article, we are reporting the case of a patient with extensive intracranial meningiomatosis who regressed completely after partial treatment with radiosurgery, likely due to abscopal effect. The patient consented to this report.

## Case presentation

**Figure 1:**
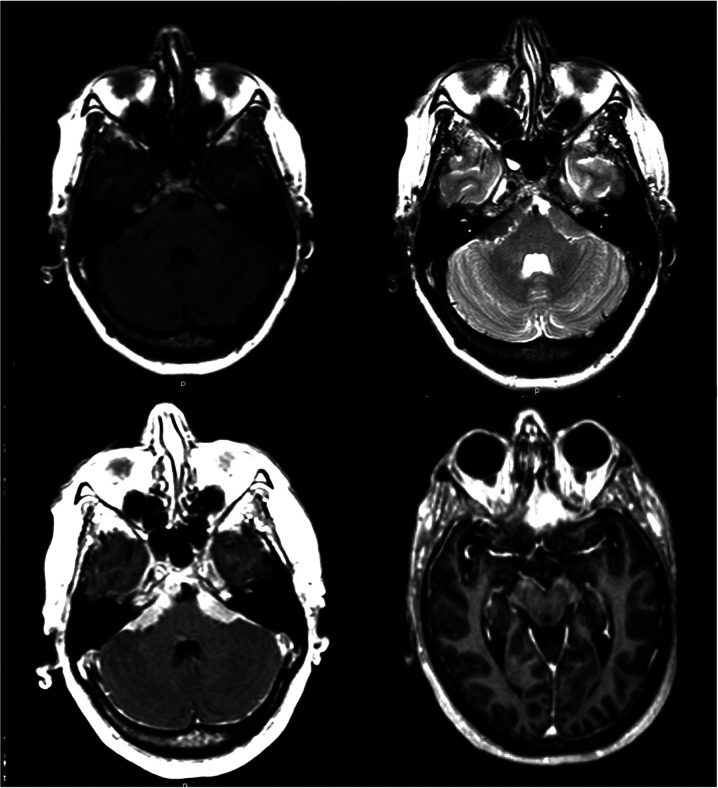
MRI sequences demonstrating typical appearance of extensive skull base meningioma (upper row left: T1 without contrast, upper row right: T2, lower row: T1 with contrast).

A 70-years-old female was referred to our department for extensive intracranial meningiomatosis involving the skull base and falx. She was known for a history of multiple venous thrombosis and was under lifetime anticoagulant therapy. She had initially presented 10 years before with subjective left side hearing loss, occipital headache, and balance problems, which recently worsened to require the use of a walker. She started to experience dysphagia in the past year. On physical examination, she had truncal ataxia and reduced hearing on the left side, with reduced gag reflex. She did not have any other focal neurologic deficit. No audiogram was available to confirm hearing loss. Her MRI showed extra-axial mass lesions involving bilateral petroclival regions, cavernous sinus, planum sphenoidale and falx with broad dural contact. Those lesions were isointense on T1 and T2/FLAIR sequences and demonstrated avid homogeneous enhancement after gadolinium injection. No brain edema was present. They had slowly progressed over many years, now causing significant brainstem compression (Figure 1). The differential diagnosis of extensive skull base tumors includes meningiomas, hemangiopericytomas, chordomas and chondrosarcomas, among others. However, those specific imaging findings combined with the long-term progression were deemed most compatible with a diagnosis of extensive *en plaque* benign meningiomas. She had previously declined whole-brain radiation therapy that was offered in another institution and did not want to attempt surgical resection or biopsy due to her comorbidities and resultant surgical high risks. She never received any corticosteroids, as there was no edema on imaging, and it was felt that chronic administration would have led to significant risks of toxicity.

**Figure 2:**
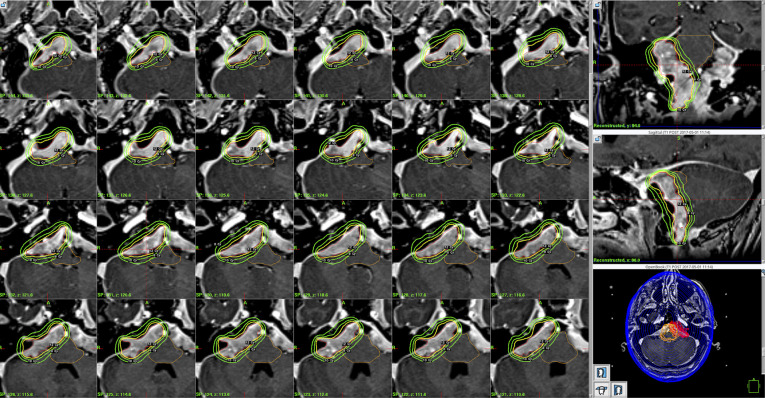
Leksell gamma plan snapshot of the radiosurgery plan.

The patient was reviewed at our multidisciplinary neuro-oncology tumor board. We elected to perform volume-staged gamma knife stereotactic radiosurgery (GKRS) instead of other conformal radiation techniques because we felt that in view of the large tumor volume and critical structure involvement, this would be the best way to minimize radiation exposure to normal neural tissue. We initially targeted the right petroclival tumor which was causing most of the brainstem compression. The goal of the procedure, as agreed with the patient, was to try to stabilize her symptoms and prevent any further deterioration. We were planning to target the left petroclival tumor 6 months later if the patient did not present any morbidity from the first session. Using Leksell Gamma Knife Perfexion (Elekta AB), a total of 41 isocenters were used to generate the treatment plan. The total treatment volume was 14.4 cc and a prescription dose of 12 Gy was delivered on the 50% isodose line (Figure 2).

**Figure 3:**
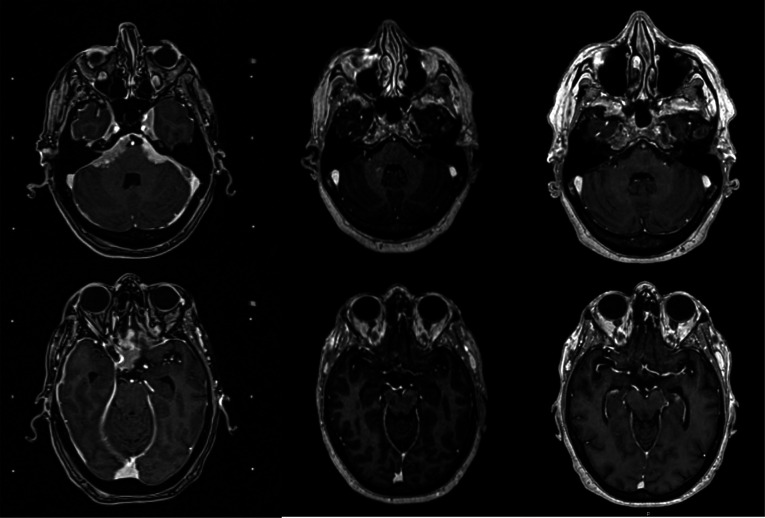
Contrast-enhanced T1 MR images at the time of radiosurgery (left column), at 6 months follow-up (middle column) and at 52 months follow-up (right column).

The clinical condition of the patient gradually improved starting at 3 months following GKRS. At 6 months, she had complete resolution of dysphagia and reduction of ataxia and headaches. At that time, a follow-up (FU) MRI demonstrated complete disappearance of all meningiomas, even outside of the radiation field. The other GKRS stages were thus cancelled, and the patient has been followed clinically and radiologically since. At 12 months, headaches had resolved. She still had mild ataxia but was walking independently without her walker. All her residual symptoms completely disappeared at 24 months after GKRS. No side effect or toxicity occurred after the procedure. At the last FU, 52 months after GKRS, the patient still has no residual symptom, walking independently without any aid, and complete tumor response is maintained on MRI (Figure 3).

## Discussion

In this article, we described a patient with complete disappearance of an extensive skull base and falx meningiomatosis after treatment of only a part of the tumor *via* GKRS. We believe the most likely explanation for this outcome is *via* the abscopal effect.

The abscopal effect is defined as a partial or complete response of a remote tumor as result of local therapy of a separate distinct tumor, most commonly radiation therapy. The dominant hypothesis is that local therapy activates a systemic immune response that then is effective against the remote lesion. The proposed immunological pathway is that radiation-induced cell death leads to antigen release from the tumor cells which then activates remote T-cells against those (4, 5). Also, radiation induces cytokines such as interferon 1 and 2 that further augment the immunological effect (6, 7). No study has demonstrated that the specific radiation technique (single fraction radiosurgery vs. fractionated) has an impact on the occurrence of abscopal effect. In a preclinical study, tumors with P53 germline mutation were more associated with the occurrence of post-radiation abscopal effect (8), so this might also be influenced by genetic predisposition.

The abscopal effect is rarely reported in CNS tumors, especially in meningiomas. This might be due to the CNS being considered as a sanctuary site escaping from the immune system. So far, there has been only one other case of suspected abscopal effect in meningioma reported in the literature (9). Golub et al. reported an 84-years old male patient who was treated with fractionated radiotherapy for an atypical meningioma (9). He also had another meningioma that was not included in the radiation treatment field. On follow-up serial annual MRI, complete response of the treated tumor was seen, along with complete regression of the untreated lesion. In this case, the abscopal effect could be postulated to be related to the atypical grade of the tumor, as higher-grade tumors might yield a stronger immune effect when exposed to radiation (10). Our case is the first one of presumed low-grade meningioma demonstrating a possible response *via* the abscopal effect after single-fraction stereotactic radiosurgery. Beside an immunologic response, other systemic factors could play a role in inducing response in untreated tumors. Meningiomas are known to have progesterone receptors which could explain spontaneous tumor regression in post-menopause women. Also, some authors have suggested that microvasculopathy could play a role in tumor rapid response in diabetic patients (11, 12). However, those mechanisms are unlikely to have been involved in our patient, who was treated well after her menopause and was not diabetic. The main limitation of our report is the lack of histopathologic confirmation of tumor histology before GKRS. However, the differential diagnosis of extensive slow-growing skull-base and falx extra-axial tumors is limited. Meningiomas are the most common, but hemangiopericytomas, mesenchymal tumors and low-grade lymphocytic and histiocytic tumors can also occur. Nevertheless, such pathologies also would not be expected to show complete disappearance of all lesions after only partial coverage *via* GKRS. As such, we believe the abscopal effect remains the most likely explanation for our patient evolution.

## Conclusion

The abscopal effect is an extremely rare phenomenon after local radiation therapy of a remote tumor. Our case demonstrates that this can occur in remote meningiomas after single fraction GKRS of a different tumor.

## Data Availability

The original contributions presented in the study are included in the article/Suplementary Material, further inquiries can be directed to the corresponding author/s.

## References

[1] Adjemian S Oltean T Martens S Wiernicki B Goossens V Vanden Berghe T Ionizing radiation results in a mixture of cellular outcomes including mitotic catastrophe, senescence, methuosis, and iron-dependant cell death. Cell Death Dis. (2020) 1003:1–15. 10.1038/s41419-020-03209-y PMC7684309 33230108

[2] Demaria S Ng B Devitt ML Babb JS Kawashima N Liebes L Ionizing radiation inhibition of distant untreated tumors (abscopal effect) is immune mediated. *Int J Radiat Oncol, Biol, Phys*. (2004) 58(3):862–70. 10.1016/j.ijrobp.2003.09.012 14967443

[3] Siva S MacManus MP Martin RF Martin OA. Abscopal effects of radiation therapy: a clinical review for the radiobiologist. Cancer Lett. (2015) 356(1):82–90. 10.1016/j.canlet.2013.09.018 24125863

[4] Sharabi AB Nirschl CJ Kochel CM Nirschl TR Francica BJ Velarde E Stereotactic radiation therapy augments antigen-specific PD-1-mediated antitumor immune responses via cross-presentation of tumor antigen. Cancer Immunol Res. (2015) 3(4):345–55. 10.1158/2326-6066.CIR-14-0196 25527358 PMC4390444

[5] Gameiro SR Jammed ML Wattenberg MM Tsang KY Ferrone S Hodge JW. Radiation-induced immunogenic modulation of tumor enhances antigen processing and calreticulin exposure, resulting in enhanced T-cell killing. Oncotarget. (2014) 5(2):403–16. 10.18632/oncotarget.1719 24480782 PMC3964216

[6] Gerber SA Sedlacek AL Cron KR Murphy SP Frelinger JG Lord EM. IFN-*γ* mediates the antitumor effects of radiation therapy in a murine colon tumor. Am J Pathol. (2013) 182(6):2345–54. 10.1016/j.ajpath.2013.02.041 23583648 PMC3668027

[7] Matsumura S Wang B Kawashima N Braunstein S Badura M Cameron TO Radiation-induced CXCL16 release by breast cancer cells attracts effector T cells. *J Immunol*. (2008) 181(5):3099–107. 10.4049/jimmunol.181.5.3099 18713980 PMC2587101

[8] Camphausen K Moses MA Ménard C Sproull M Beecken WD Folkman J, Radiation abscopal antitumor effect is mediated through p53. Cancer Res. (2003) 63(8):1990–3 12702593

[9] Golub D Kwan K Knisely JPS Schulder M. Possible abscopal effect observed in frontal meningioma after localized IMRT on posterior meningioma resection cavity without adjuvant immunotherapy. Front Oncol. (2019 Oct 18) 9:1109. 10.3389/fonc.2019.01109 31681619 PMC6813201

[10] Kosugi K Tamura R Ohara K Morimoto Y Kuranari Y Oishi Y Immunological and vascular characteristics in cavernous sinus meningioma. J Clin Neurosci. (2019) 67:198–203. 10.1016/j.jocn.2019.06.003 31213381

[11] Kalamarides M Peyre M. Dramatic shrinkage with reduced vascularization of large meningiomas after cessation of progestin treatment. World Neurosurg. (2017) 101:814.e7–e10. 10.1016/j.wneu.2017.03.013 28300711

[12] Wahab M Al-Azzawi F. Meningioma and hormonal influences. Climacteric. (2003) 6(4):285–92. 10.1080/cmt.6.4.285.292 15006250

